# Worse cardiovascular prognosis after endovascular surgery for intermittent claudication caused by infrainguinal atherosclerotic disease in patients with diabetes

**DOI:** 10.1177/2042018820960294

**Published:** 2020-10-19

**Authors:** Ardwan Dakhel, Moncef Zarrouk, Jan Ekelund, Stefan Acosta, Peter Nilsson, Mervete Miftaraj, Björn Eliasson, Ann-Marie Svensson, Anders Gottsäter

**Affiliations:** Department of Vascular Diseases, Lund University, Skåne University Hospital, Malmö, S-205 02, Sweden; Department of Vascular Diseases, Lund University, Skåne University Hospital, Malmö, Sweden; Swedish National Diabetes Register, Gothenburg, Sweden; Department of Vascular Diseases, Lund University, Skåne University Hospital, Malmö, Sweden; Department of Internal Medicine, Clinical Research Unit, Lund University, Skåne University Hospital, Malmö, Sweden; Swedish National Diabetes Register, Gothenburg, Sweden; Swedish National Diabetes Register, Gothenburg, Sweden; Department of Molecular and Clinical Medicine, Institute of Medicine, University of Gothenburg, Gothenburg, Sweden; Swedish National Diabetes Register, Gothenburg, Sweden; Department of Vascular Diseases, Lund University, Skåne University Hospital, Malmö, Sweden

**Keywords:** diabetes mellitus, endovascular surgery, intermittent claudication, long-term follow-up, peripheral arterial disease

## Abstract

**Background:**

Diabetes mellitus (DM) is an established risk factor for intermittent claudication (IC) and other manifestations of atherosclerotic peripheral arterial disease. Indications for surgery in infrainguinal IC are debated, and there are conflicting reports regarding its outcomes in patients with DM. Aims of this study were to compare both short- and long-term effects on total- and cardiovascular (CV) mortality, major adverse cardiovascular events (MACEs), acute myocardial infarction (AMI), stroke, and major amputation following infrainguinal endovascular surgery for IC in patients with and without DM. We also evaluated potential relationships between diabetic control and outcomes in patients with DM.

**Methods:**

Nationwide observational cohort study of patients registered in the Swedish Vascular Registry and the Swedish National Diabetes Registry. Propensity score adjusted comparison of total and CV mortality, MACE, AMI, stroke, and major amputation after elective infrainguinal endovascular surgery for IC in 626 patients with and 1112 without DM at 30 postoperative days and after median 5.2 [interquartile range (IQR) 4.2–6.3] years of follow-up for patients with DM, and 5.4 (IQR 4.3–6.5) years for those without.

**Results:**

In propensity score adjusted Cox regression after 30 postoperative days, there were no differences between groups in morbidity or mortality. At last follow-up, patients with DM showed higher rates of MACE [hazard ratio (HR) 1.26, confidence interval (CI) 1.07–1.48; *p* < 0.01], AMI (HR 1.48, CI 1.09–2.00; *p* = 0.01), and major amputation (HR 2.31, CI 1.24–4.32; *p* < 0.01). Among patients with DM, higher HbA1c was associated with higher total mortality during follow-up (HR 1.01, CI 1.00–1.03; *p* = 0.045).

**Conclusion:**

Patients with DM have higher rates of MACE, AMI, and major amputation in propensity score adjusted analysis during 5 years of follow-up after infrainguinal endovascular surgery for IC. Furthermore, HbA1c is associated with total mortality in patients with DM. Prevention and treatment of DM is important to improve cardiovascular and limb outcomes.

## Introduction

Intermittent claudication (IC) is a common manifestation of atherosclerotic peripheral arterial disease (PAD), manifesting itself as pain in the lower extremities induced by exercise and relieved by rest.^1^ IC is estimated to affect 20–40 million individuals worldwide,^2^ and its prevalence in Sweden is 6.6% in women and 7.2% in men aged 60–90 years.^3^ Diabetes mellitus (DM), arterial hypertension, hypercholesterolaemia, and smoking are all well-established risk factors for IC, as well as for other forms of PAD and atherosclerotic cardiovascular (CV) disease (CVD).^2^

The strong associations between DM and the development of both infrainguinal PAD^4^ and other forms of CVD are due to the pro-atherogenic effects of the diabetic state.^5,6^ By increasing vascular inflammation and dyslipidaemia, and by leading to an overproduction of advanced glycation end-products, DM causes increased endothelial dysfunction.^6^ In addition, PAD patients with DM often have more distally located atherosclerotic lesions and experience worse lower extremity function than those with PAD alone.^7,8^

IC can be treated electively with endovascular or open surgery. Endovascular surgery with percutaneous balloon angioplasty and a variety of other devices is increasingly used as it is minimally invasive, requires shorter hospital stay, and has lower postoperative complication rates.^1,9^ For IC patients with symptoms caused by infrainguinal atherosclerotic lesions, however, indications for revascularization are limited.^1^ Invasive treatment is recommended only for those with lifestyle limiting claudication not responding to guideline-directed medical and exercise therapy.^1,9^ In such cases, endovascular surgery is recommended as first choice for patients with lesions up to 25 cm in length regardless of the presence of DM.^1^

Previous studies^4,10,11^ have shown varying results regarding the impact of DM on mortality and amputations after endovascular surgery for IC, and data on other vascular outcomes such as acute myocardial infarction (AMI) and stroke have been scarcely reported. Shammas *et al.* found no increased mortality among patients with DM after supra- and infrainguinal endovascular surgery,^11^ whereas diabetes was associated with an increased rate of major amputation.^11^ Rizzo *et al.* reported no significant differences in either mortality or major amputation between patients with and without DM after endovascular surgery of the superficial femoral artery (SFA).^10^ Neupane and co-workers, on the other hand, reported increased risks for both all-cause mortality and major amputation in patients with DM following popliteal and infrapopliteal endovascular surgery. Their study also included patients with chronic limb threatening ischaemia (CLTI), however, a condition in which the indication for surgery is undebatable.^1^

The primary aim of this study was to compare propensity score-adjusted short- (30-day) and long-term total and CV mortality, MACE, AMI, stroke, and major amputation after elective endovascular surgery for infrainguinal IC in patients with and without DM. The secondary aim was to evaluate potential relationships between glycaemic control and outcomes in patients with DM.

## Methods

### Databases and procedures

We linked information from Swedish nationwide population-based databases using the 12-digit personal identity number unique to every Swedish inhabitant. Subjects were identified in the Swedish Vascular Registry (Swedvasc),^12^ and the Swedish National Diabetes Registry (NDR).^13^ Swedvasc comprises data on all patients undergoing invasive vascular treatment at Swedish hospitals, and has an external validity of 93% for infrainguinal procedures.^12^ Preoperative risk data are registered together with the type of treatment (acute or elective, open, endovascular, or hybrid surgery). Patients are followed up at 1 and 12 months after each surgical procedure. NDR is a nationwide quality assurance and improvement tool for care providers, covering the majority of Swedish diabetic patients aged 18 years or older. It contains data about clinical characteristics, risk factors, diabetes-related complications, and treatments.

**Figure 1.**
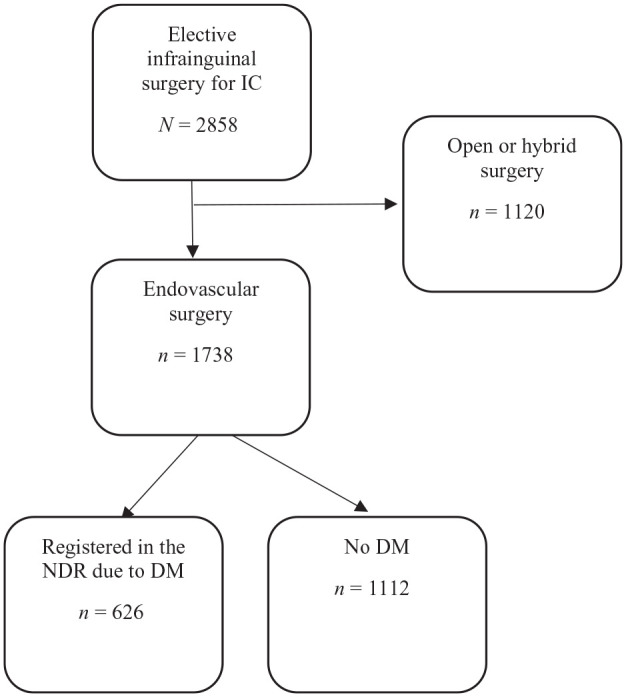
Flow chart of patients in the Swedish Vascular Registry (Swedvasc) undergoing elective infrainguinal surgery for intermittent claudication (IC) during 2010–2014, with and without registration in the National Diabetes Registry (NDR) with a diagnosis of diabetes mellitus (DM).

In this study, patients registered in the Swedvasc infrainguinal module after elective endovascular surgery from 2010 to 2014 were identified, and those with a concomitant registration in the NDR (diabetic group) were compared with those without such registration (non-diabetic group; Figure 1).

### Baseline data

Baseline data were retrieved from several other registries. The Inpatient Register (IPR) administered by the National Board of Health and Welfare (http://socialstyrelsen.se/english)^14^ with nationwide data for primary and secondary discharge diagnoses and lengths of hospitalization since 1987,^14^ the Prescribed Drug Register (PDR) with complete information about filled prescriptions since 2005,^15^ and the cancer registry^16^ were used for information about comorbidities and drug treatment at baseline.

The IPR uses International Classification of Diseases (ICD) revision 10, for classification of diagnoses. Comorbidities at baseline included: heart failure (HF), atrial fibrillation or flutter (AF), hypertension, coronary heart disease (CHD), and stroke. AMI was defined as I21 (ICD-10) and CVD as CHD and/or stroke prior to index date. At baseline, we also included psychiatric disorders, dementia, cancer, and renal disorders. All ICD codes used in the study are listed in Supplemental Appendix 1.

The PDR helped us define drug treatment at baseline. Use of acetylsalicylic acid (ASA), lipid-lowering medications and anticoagulant therapy was included. Hypertension was defined as three or more prescriptions for antihypertensive medication for 1 year prior to index operation date. One prescription is equivalent to 3 months of continuous use of a drug. The most common antihypertensive medication in Sweden at the time of the study were: angiotensin-converting-enzyme (ACE) inhibitors, diuretics, calcium channel blockers, angiotensin II-receptor blockers, alpha-1 receptor blockers, beta blockers, and various combinations of the above. Use of lipid-lowering drugs and ASA was defined in a similar manner.

The longitudinal integration database for health insurance and job market studies (LISA; Statistics Sweden) was used to obtain socioeconomic characteristics. Marital status was broken down into: single, married, divorced or widowed. Educational level was broken down into compulsory school, upper secondary school, and college or university.

### Follow-up

Total and CV mortality was followed-up until 31 December 2017, using the Cause of Death Register with complete information on the causes and time of death,^17^ administered by the National Board of Health and Welfare (http://socialstyrelsen.se/english). The IPR^14^ was used for follow-up of MACEs (defined as ischaemic heart disease defined as ICD codes I20–I25, cerebrovascular disease defined as ICD codes I61–I64, and coronary revascularization defined as procedure codes FN),^18^AMI, stroke, and major amputation.

### Statistical analysis

Total and CV mortality, MACE, AMI, stroke, and major amputations after elective endovascular surgery for infrainguinal IC were compared between patients with and without DM at 30 postoperative days and at end of follow-up. The analyses were propensity score adjusted, using Cox regression analysis for the following variables: age, gender, smoking, previous AMI, CHD, stroke, cerebrovascular disease, AF, HF, renal disease, malignant disease, liver disorders, psychiatric disorders, peripheral arterial disease, chronic obstructive pulmonary disease, lipid- and blood pressure-lowering drugs, ASA, anticoagulation, ACE-inhibitors, angiotensin receptor-, alpha-, beta-, and calcium channel blockers, diuretics, digoxin, yearly income, civil status, country of birth, and education.

Propensity scores were estimated using a generalized boosted multinomial regression model with an interaction depth of 3, a maximum of 10,000 trees, and a shrinkage of 0.01. The optimal number of trees was selected using a stopping rule applied to the degree of balance. Descriptive statistics is presented using mean, standard deviation, median, interquartile range (IQR), counts, and percentages with 95% confidence intervals (CIs) according to variable type. The degree of similarity between infrainguinal IC patients with and without DM is described using standardized mean difference. Incidence rates for total and CV mortality, MACE, AMI, stroke, major amputation, and a composite of death or amputation are estimated as the number of events per 1000 person-years with exact 95% Poisson CIs. Cumulative total and CV mortality, MACE, AMI, stroke, major amputation, and a composite of death or major amputation are described using Kaplan–Meier curves transformed to estimate distribution function rather than survival function. The analyses compare infrainguinal IC patients with DM with infrainguinal IC patients without DM using both unadjusted and inverse probability of treatment weighting (IPTW) adjusted Cox regression presented as hazard ratio (HR). *p*-values < 0.05 were considered significant. Most variables were derived from mandatory health data or population registries and therefore have virtually no missing values, except smoking, for which approximately 15% of data was missing. The gradient boosting machine model used in the estimation of the propensity score treats missing values as a separate category and attempts to balance the proportion of missing values as well as non-missing values. Analyses were performed using R 3.4.3 (http://cran.us.r-project.org/).

### Ethical approval

The study was approved by The Regional Board for Research Ethics in Lund, Sweden (2016/232 and 2016/544). All patients had given their written informed consent to registration in Swedvasc and NDR.

## Results

### Study population and demographic characteristics

Of 2858 patients registered in Swedvasc after an elective infrainguinal intervention for IC during 2010–2014, 1738 (67.2%) had been treated with endovascular surgery. In this group the 626/1738 (36.0%) that were registered in NDR due to DM were compared with the remaining 1112/1738 (64.0%) without DM (Figure 1). The majority of patients with DM [575 (91.9%)] were classified as having type 2 DM, 47 (7.5%) as having type 1, and three (0.6%) as having other or unspecified types of DM. Median follow-up was 5.2 (IQR 4.2–6.3) years for patients with DM and 5.4 (IQR 4.3–6.5) for patients without. Table 1 presents complete unadjusted baseline data, clinical, and demographic characteristics of the two groups.

**Table 1.** Baseline characteristics of patients without and patients with diabetes mellitus undergoing elective infrainguinal endovascular surgery for intermittent claudication.

	No diabetes *n* = 1112	Diabetes *n* = 626	Test	SMD
Age, years [mean (SD)]	73.35 (8.84)	70.59 (8.46)		0.319
Female gender [*n* (%)]	591 (53.1)	233 (37.2)		0.324
Smoking [*n* (%)]	127 (13.4)	75 (12.9)		0.015
Duration of DM, years	ND	13.56 (11.48)		
HbA1c, mmol/mol	ND	59.75 (15.00)		
Medication [*n* (%)]				
Lipid lowering	882 (79.3)	565 (90.3)		0.308
Antihypertensive	919 (82.6)	588 (93.9)		0.356
Aspirin	898 (80.8)	526 (84.0)		0.086
Anticoagulant	292 (26.3)	204 (32.6)		0.139
ACE-inhibitor	364 (32.7)	291 (46.5)		0.284
ARB	285 (25.6)	240 (38.3)		0.275
Alpha blocker	18 (1.6)	20 (3.2)		0.103
Beta blocker	504 (45.3)	368 (58.8)		0.272
CCB	446 (40.1)	325 (51.9)		0.239
Diuretic	382 (34.4)	275 (43.9)		0.197
Digoxin	22 (2.0)	20 (3.2)		0.077
Nitrates	203 (18.3)	153 (24.4)		0.151
Monthly income, USD [mean (SD)]	1911,13 (1501.09)	1950,41 (2092.01)		0.022
Education [*n* (%)]				0.0079
Compulsory	451 (42.2)	259 (42.2)		
Upper secondary	472 (42.8)	284 (46.3)		
College or university	71 (11.6)	71 (11.6)		
Civil status [*n* (%)]				0.228
Married	514 (46.2)	328 (52.4)		
Separated	259 (23.3)	144 (23.0)		
Single	89 (8.0)	53 (8.5)		
Widowed	250 (22.5)	101 (16.1)		
Origin [*n* (%)]				0.232
Europe except Sweden	88 (7.9)	59 (9.4)		
Rest of the world	67 (6.0)	77 (12.3)		
Sweden	957 (86.1)	490 (78.3)		
Previous diseases [*n* (%)]				
AMI	137 (12.3)	125 (20.0)		0.209
Coronary heart disease	318 (28.6)	283 (45.3)		0.349
Stroke	79 (7.1)	69 (11.0)		0.137
Cerebrovascular disease	204 (18.3)	179 (28.0)		0.229
Atrial fibrillation	109 (9.8)	78 (12.5)		0.085
Congestive heart failure	93 (8.4)	81 (12.9)		0.149
Renal disorder	41 (3.7)	39 (6.2)		0.117
Liver disease	6 (0.5)	5 (0.8)		0.032
Psychiatric disorders	28 (2.5)	20 (3.2)		0.041
COPD	108 (9.7)	52 (8.3)		0.049

ACE, angiotensin converting enzyme; AMI, acute myocardial infarction; ARB, angiotensin receptor blocker; CCB, calcium channel blocker; COPD, chronic obstructive pulmonary disease; USD, United States Dollars; DM, diabetes mellitus; SMD, standardized mean difference.

### Follow-up

In the IPTW-adjusted Cox regression, there were no differences between patients with and patients without DM in total or CV mortality during the first 30 postoperative days (Table 2). Neither did the risk of postoperative MACE, AMI, stroke, and major amputation differ between groups.

**Table 2.** Propensity score adjusted analyses after the first 30 postoperative days and after 5.2–5.4 years of follow-up after elective infrainguinal endovascular surgery for intermittent claudication in patients with (*n* = 626) diabetes mellitus (DM) compared with patients without DM (*n* = 1112).

	HR	*p-*value	95% CI
**After 30 days**			
Mortality	0.86	0.8470	0.19–3.89
CV mortality	0.77	0.7795	0.13–4.66
MACE	1.06	0.7703	0.73–1.52
AMI	1.68	0.3364	0.58–4.86
Stroke	0.38	0.3844	0.04–3.39
Major amputation	1.80	0.5613	0.25–13.23
Major amputation or death	0.91	0.8619	0.32–2.60
**After 5.2–5.4 years**			
Mortality	1.12	0.2901	0.91–1.40
CV mortality	1.14	0.3225	0.88–1.49
MACE	1.26	0.0051	1.07–1.48
AMI	1.48	0.0113	1.09–2.00
Stroke	1.25	0.2515	0.86–1.81
Major amputation	2.31	0.0087	1.24–4.32
Major amputation or death	1.18	0.1336	0.95–1.45

Hazard ratio (HR) for total and cardiovascular (CV) mortality, major adverse CV events (MACEs), acute myocardial infarction (AMI), stroke, major amputation, and the composite of major amputation and death; *p*-values and 95% confidence intervals (CIs).

During long-term follow-up, the IPTW-adjusted Cox regression (Table 2) revealed that patients with DM had higher rates of MACE (HR 1.26, CI 1.07**–**1.48; *p* < 0.01), AMI (HR 1.48, CI 1.09–2.00; *p* = 0.01) and major amputation (HR 2.31, CI 1.24–4.32; *p* < 0.01). Incidence rates are shown in Supplemental Appendix 2.

**Figure 2.**
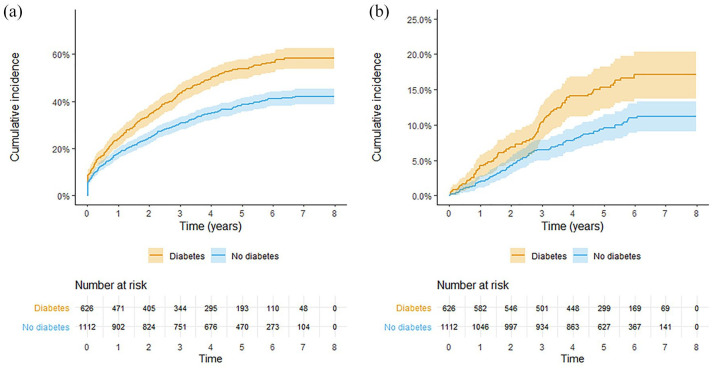
Crude Kaplan–Meier curves showing cumulative incidence (with 95% confidence intervals) of major cardiovascular events (a) and acute myocardial infarction (b) after elective endovascular surgery for infrainguinal intermittent claudication in patients with and patients without diabetes mellitus.

Kaplan–Meier curves of cumulative incidences of MACE and AMI are shown in Figure 2. Among patients with DM, higher HbA1c was associated with higher risk of total mortality during follow-up (HR 1.01, CI 1.00–1.03; *p* = 0.0453), whereas neither duration of diabetes nor HbA1c level was related to CV mortality, MACE, AMI, stroke, or major amputation during follow-up (Supplemental Appendix 3).

## Discussion

Propensity score adjusted analyses showed no differences between groups concerning total or CV mortality, MACE, AMI, stroke, or major amputation during the first 30 postoperative days, whereas patients with DM had a significantly higher risk of MACE, AMI, and major amputation during long-term follow-up. There were no differences between groups concerning total or CV mortality, or stroke, however, after adjustment for baseline differences.

Baubeta Fridh and co-workers analysed mortality and amputation rates during 3 years in a heterogeneous group of PAD patients in Swedvasc having undergone either open or endovascular surgery for CLTI or IC caused by supra- or infrainguinal disease.^19^ Mortality in the IC group was 3.4% after 1 year and 12.0% after 3 years. Their 1 year mortality rate is comparable to ours despite our more narrow inclusion criteria, whereas the 5 year mortality in our study cannot be directly compared with their figures due to their shorter follow-up.^19^ As amputation rates in IC patients in the study by Baubeta Fridh and co-workers^19^ were initially low and increased linearly over time, the authors speculated that amputations were not procedure related, supporting the notion that revascularization might still be a viable option in IC. Similarly, we report no significant difference between the groups in neither mortality nor amputations during the initial 30 postoperative days, whereas the well-established risk for CV morbidity and mortality in DM^20,21^ was reflected in the long-term results, showing increased rates of MACE and AMI in patients with DM. Hence, the increased rate of CV events might be due to the underlying macrovascular disease, accelerated microcirculatory disturbances, and diabetic peripheral neuropathy,^5,6,22^ rather than to procedure related complications.

Whether outcomes are more unfavourable in patients with DM undergoing endovascular surgery for IC has previously been debated,^5,6,22^ but previously reported materials are either smaller or have shorter follow-up than in the present study. Furthermore, the patient groups have not always been strictly selected with regard to symptoms, level of atherosclerotic occlusive lesions, or operative technique. The recent retrospective study^10^ by Rizzo *et al.* compared the outcomes after SFA revascularization in a small study of 110 patients with symptomatic PAD, 52 of whom suffered from IC. During a median follow-up of 18 months, no statistically significant differences in mortality were observed between patients with and patients without DM. The authors speculate that this may be due to other group differences in spite of adjustment, such as patient and disease characteristics.^10^ In another retrospective study^11^ based on the Excellence in Peripheral Artery Disease registry, Shammas *et al.* analysed outcomes following both supra- and infrainguinal endovascular surgery in patients with and patients without DM. Of the 1906 patients with symptomatic PAD enrolled, 1198 presented with IC. During the 12 month follow-up, IC patients with DM showed higher rates of major amputation, whereas there was no significant difference in mortality between groups.^11^ These results are comparable to ours, but follow-up was shorter and the risk of MACE, AMI, or stroke was not assessed. The retrospective single-centre study by Neupane *et al.* evaluated outcomes of 714 patients undergoing popliteal and infrapopliteal endovascular surgery for CLTI or IC.^4^ During the median 3 years of follow-up, patients with DM showed higher rates of both all-cause mortality and major amputation.

In current guidelines^1^ the presence of DM is not specifically taken into account when recommending endovascular rather than open surgery as first choice in infrainguinal IC for patients with lifestyle limiting claudication in spite of medical and exercise therapy.^22^ To minimize the risk of confounding and selection bias, we restricted our analysis to IC patients treated with endovascular methods. It could not be excluded, however, that the subgroup with DM in whom endovascular methods were chosen had more or less complex atherosclerotic disease than those without DM. Relevant comorbidities besides DM that might have influenced the long-term outcome following endovascular surgery in our study were fully adjusted for in the propensity score analysis. Comorbidities were entered as categorical instead of continuous variables, and dependent on the accuracy of the IPR.

Patients with DM are at higher risk of CVD and other diabetes related complications and are therefore recommended aggressive management of CV risk factors.^23^ Consequently, the proportion of patients using cardioprotective drugs in our study was higher in the group with DM despite the fact that current guidelines^1^ in fact recommend such medications in all patients with PAD. These differences were adjusted for by the propensity scoring, but as we did not assess lipid or blood pressure levels, we cannot exclude that differences in these risk factors might have affected our results.

In the present study patients with DM and elevated perioperative HbA1c levels showed higher total mortality, but no significantly increased risk of cardiovascular morbidity or amputation. Results of our study differ from a previous analysis^24,25^ of a large heterogeneous material of 26,799 patients with CLTI and IC caused by supra- and infrainguinal PAD undergoing both open and endovascular surgery. Increased HbA1c was associated with increased rates of major amputation during a median follow-up of 3.8 years, whereas total mortality was not assessed.^25^ In the study of Neupane *et al.*,^4^ on the other hand, HbA1c levels were related to neither mortality nor major amputation, and there was no difference in outcome between insulin- and non-insulin-requiring patients. Potential relationships between microvascular diabetic complications and events^26,27^ in the group with DM have not been evaluated in the present study.

In this context, it should be emphasized that results in an IC population should not be compared with those in a population partly consisting of CLTI patients. Furthermore, outcomes after different surgical procedures performed at different anatomical locations should not be directly compared.

The major strength of the present nationwide study is the comparably long follow-up of over 5 years and the large number of patients followed after infrainguinal endovascular surgery. Our study also had several imitations other than its retrospective design. As there is a possible correlation between insulin dependence and increased risk of amputation in patients with CLTI undergoing endovascular surgery,^28^ separate analyses of insulin- and non-insulin-treated patients would have been desirable. During the 5.2 year follow-up period novel antidiabetic medications have been introduced, which have shown to improve the overall cardiovascular risk profile.^29^ As we have not accounted for the antidiabetic medications in our study, potential effects of the antidiabetic therapy upon our outcomes cannot be separated from the effects of diabetes in itself. It would also have been desirable to analyse precise location and extent of the atherosclerotic lesions according to the Trans-Atlantic Inter-Society Consensus II classification,^30^ as previous studies have implicated a correlation between lesion extent and risk of amputation.^10^ Due to the risk of misclassification of CLTI as IC^31^ and uncertainty of amputation data in patient registries, the reported association between diabetes and increased risk of major amputation should be interpreted very cautiously. Unfortunately, we did not have ethical permission to validate amputation data in individual patient files at different centres.

Invasive treatment of infrainguinal IC is recommended only for those with lifestyle limiting disease,^1^ and has been proven to improve health related quality of life (HRQoL) for up to 24 months of follow-up.^32^ In a mixed cohort of patients with supra- and infrainguinal IC such benefits were no longer detectable after 5 years,^33^ however, reflecting the uncertain indication for surgery in IC. Data concerning HRQoL were unfortunately not available in our study. The worse long-term prognosis in patients with DM in our study underscores the importance of extra careful ascertainment of potential HRQoL gains when considering patients with DM and infrainguinal IC for endovascular surgery.

## Conclusion

This nationwide, observational, longitudinal propensity score adjusted study demonstrated no significant increase in procedure related events in patients with DM after infrainguinal endovascular surgery for IC, whereas patients with DM had higher rates of AMI, MACE, and major amputation during 5 years of follow-up. HbA1c was associated with total mortality in diabetic patients. Prevention and treatment of DM is important to improve cardiovascular and limb outcomes.

## Supplemental Material

sj-docx-1-tae-10.1177_2042018820960294.docx – Supplemental material for Worse cardiovascular prognosis after endovascular surgery for intermittent claudication caused by infrainguinal atherosclerotic disease in patients with diabetes Click here for additional data file. Supplemental material, sj-docx-1-tae-10.1177_2042018820960294.docx for Worse cardiovascular prognosis after endovascular surgery for intermittent claudication caused by infrainguinal atherosclerotic disease in patients with diabetes by Ardwan Dakhel, Moncef Zarrouk, Jan Ekelund, Stefan Acosta, Peter Nilsson, Mervete Miftaraj, Björn Eliasson, Ann-Marie Svensson and Anders Gottsäter in Therapeutic Advances in Endocrinology and Metabolism

sj-docx-2-tae-10.1177_2042018820960294.docx – Supplemental material for Worse cardiovascular prognosis after endovascular surgery for intermittent claudication caused by infrainguinal atherosclerotic disease in patients with diabetes Click here for additional data file. Supplemental material, sj-docx-2-tae-10.1177_2042018820960294.docx for Worse cardiovascular prognosis after endovascular surgery for intermittent claudication caused by infrainguinal atherosclerotic disease in patients with diabetes by Ardwan Dakhel, Moncef Zarrouk, Jan Ekelund, Stefan Acosta, Peter Nilsson, Mervete Miftaraj, Björn Eliasson, Ann-Marie Svensson and Anders Gottsäter in Therapeutic Advances in Endocrinology and Metabolism

sj-docx-3-tae-10.1177_2042018820960294.docx – Supplemental material for Worse cardiovascular prognosis after endovascular surgery for intermittent claudication caused by infrainguinal atherosclerotic disease in patients with diabetes Click here for additional data file. Supplemental material, sj-docx-3-tae-10.1177_2042018820960294.docx for Worse cardiovascular prognosis after endovascular surgery for intermittent claudication caused by infrainguinal atherosclerotic disease in patients with diabetes by Ardwan Dakhel, Moncef Zarrouk, Jan Ekelund, Stefan Acosta, Peter Nilsson, Mervete Miftaraj, Björn Eliasson, Ann-Marie Svensson and Anders Gottsäter in Therapeutic Advances in Endocrinology and Metabolism
