# Supplementary material for: Worse cardiovascular prognosis after endovascular surgery for intermittent claudication caused by infrainguinal atherosclerotic disease in patients with diabetes
Source: Ther Adv Endocrinol Metab. 2020 Oct 19;11:2042018820960294. doi: 10.1177/2042018820960294 (PMC7580142; doi:10.1177/2042018820960294)
Supplement: sj-docx-1-tae-10.1177_2042018820960294.docx – Supplemental material for Worse cardiovascular prognosis after endovascular surgery for intermittent claudication caused by infrainguinal atherosclerotic disease in patients with diabetes [file sj-docx-1-tae-10.1177_2042018820960294.docx.docx]

| **Appendix 1.** Concomitant diseases at baseline, ICD-10 codes. | | |
| --- | --- | --- |
| Acute myocardial infarction | I21…, |  |
| Coronary heart disease | I20…, I21…, I22…, I23…, I24…, I25…, |  |
| Stroke | I61…, I62…, I63…, I64…, |  |
| Cerebrovascular disease | I21…, I61…, I62…, I63…, I64…, |  |
| Atrial fibrillation | I48…, |  |
| Congestive heart failure | I50…, |  |
| Renal disorders | N17…, N18…, N19…, N99…, V42A, V45B, V56A, V56W, Z940, Z491, Z492, Z992 |  |
| Malignant disease | C0…, C1…, C2…, C3…, C4…, C5…, C6…, C7…, C8…, C9…, |  |
| Liver disorders | K70…, K71…, K72…, K73…, K74…, |  |
| Psychiatric disorders | F20…, F21…, F22…, F23…, F24…, F25…, F26…, F27…, F28…, F29…, F30…, F31…, F32…, F33…, F34…, F35…, F36…, F37…, F38…, F39… |  |
| Chronic obstructive pulmonary disease | J44…, |  |
