# Supplementary material for: Worse cardiovascular prognosis after endovascular surgery for intermittent claudication caused by infrainguinal atherosclerotic disease in patients with diabetes
Source: Ther Adv Endocrinol Metab. 2020 Oct 19;11:2042018820960294. doi: 10.1177/2042018820960294 (PMC7580142; doi:10.1177/2042018820960294)
Supplement: sj-docx-2-tae-10.1177_2042018820960294.docx – Supplemental material for Worse cardiovascular prognosis after endovascular surgery for intermittent claudication caused by infrainguinal atherosclerotic disease in patients with diabetes [file sj-docx-2-tae-10.1177_2042018820960294.docx.docx]

**Appendix 2.** Number of events and incidence rates (IR) per 1,000 person years with 95% Poisson confidence intervals (CI) of total and cardiovascular (CV) mortality, major adverse CV events (MACE), acute myocardial infarction (AMI), stroke, major amputation, and the composite of major amputation and death during follow-up of patients without (n=1,112), and with (n=626) type 2 diabetes mellitus after infrainguinal elective endovascular surgery for intermittent claudication.

|  |  |  |  |
| --- | --- | --- | --- |

| **Event** | **No diabetes** | **Diabetes** |
| --- | --- | --- |
| Total mortality | 261 (IR 45.0 [39.7 – 50.8]) | 171 (IR 53.7 [46.0 – 62.8]) |
| CV mortality | 158 (IR 27.2 [23.1 – 31.8]) | 110 (IR 34.5 [28.4 – 41.6]) |
| MACE | 434 (IR 96.2[87.3 – 105.7]) | 342 (IR 159.5 [143.0 – 177.3]) |
| AMI | 104 (IR 18.8[15.3 – 22.8]) | 92 (IR 31.2 [25.2 – 38.3]) |
| Stroke  Major amputation  Major amputation or death | 77 (IR 13.7 [10.8 – 17.1])  21 (IR 3.6[2.3 – 5.6])  267 (IR 46.3 [41.0 – 52.3]) | 56 (IR 18.5[13.9 – 24.0])  26 (IR 8.3[5.4 – 12.2])  181 (IR 57.8[49.7 – 66.8]) |
