# Supplementary material for: Worse cardiovascular prognosis after endovascular surgery for intermittent claudication caused by infrainguinal atherosclerotic disease in patients with diabetes
Source: Ther Adv Endocrinol Metab. 2020 Oct 19;11:2042018820960294. doi: 10.1177/2042018820960294 (PMC7580142; doi:10.1177/2042018820960294)
Supplement: sj-docx-3-tae-10.1177_2042018820960294.docx – Supplemental material for Worse cardiovascular prognosis after endovascular surgery for intermittent claudication caused by infrainguinal atherosclerotic disease in patients with diabetes [file sj-docx-3-tae-10.1177_2042018820960294.docx.docx]

**Appendix 3** Relations between glycemic control (HbA1c = glycated haemoglobin) and diabetes duration, total and cardiovascular (CV) mortality, major adverse CV events (MACE), acute myocardial infarction (AMI), stroke, major amputation, and the composite of major amputation and death during 5.2 years of follow-up after planned infrainguinal endovascular surgery for intermittent claudication in 626 patients with diabetes mellitus. Hazard ratio (HR), p-values and 95% confidence interval (CI).

| **HbA1c** | HR | **p-value** | **95% CI** |
| --- | --- | --- | --- |
| Total mortality | 1.01 | 0.0453 | 1.00 – 1.03 |
| CV mortality | 1.01 | 0.0543 | 1.00 – 1.03 |
| MACE | 1.01 | 0.0669 | 1.00 – 1.02 |
| AMI | 1.01 | 0.3299 | 0.99 – 1.03 |
| Stroke | 1.01 | 0.1915 | 0.99 – 1.04 |
| Major amputation | 1.02 | 0.1405 | 0.99 – 1.06 |
